# Wooden foreign body missed on imaging

**DOI:** 10.1002/jhm.70472

**Published:** 2026-09-09

**Authors:** Ryan Ajgaonkar, Terry J. S. Lund, Nilam J. Soni

**Affiliations:** ^1^ Division of Hospital Medicine, Joe R. Theresa Lozano Long School of Medicine University of Texas Health San Antonio San Antonio Texas USA; ^2^ Medicine Service, South Texas Veterans Health Care System San Antonio Texas USA

## CASE DESCRIPTION

A 69‐year‐old man with diabetes presented with severe left foot pain after “stepping on something sharp.” Striking erythema of the left foot was noted with 2+ pitting edema extending proximally to the tibial tuberosity (Figure 1). On the plantar surface near the second metatarsophalangeal joint, a 1.5 mm puncture wound without drainage was identified. Computed tomography (CT) and magnetic resonance imaging (MRI) were negative for foreign body; however, a point‐of‐care ultrasound exam demonstrated a prominent vertical shadow at the puncture site, suggesting a retained foreign body (Figure 2, Video 1). After minimal improvement on intravenous antibiotics, surgical exploration of the wound was performed and revealed a 1.8 cm wooden toothpick (Figure 3). The patient's cellulitis resolved after a course of oral antibiotics.

**Figure 1:**
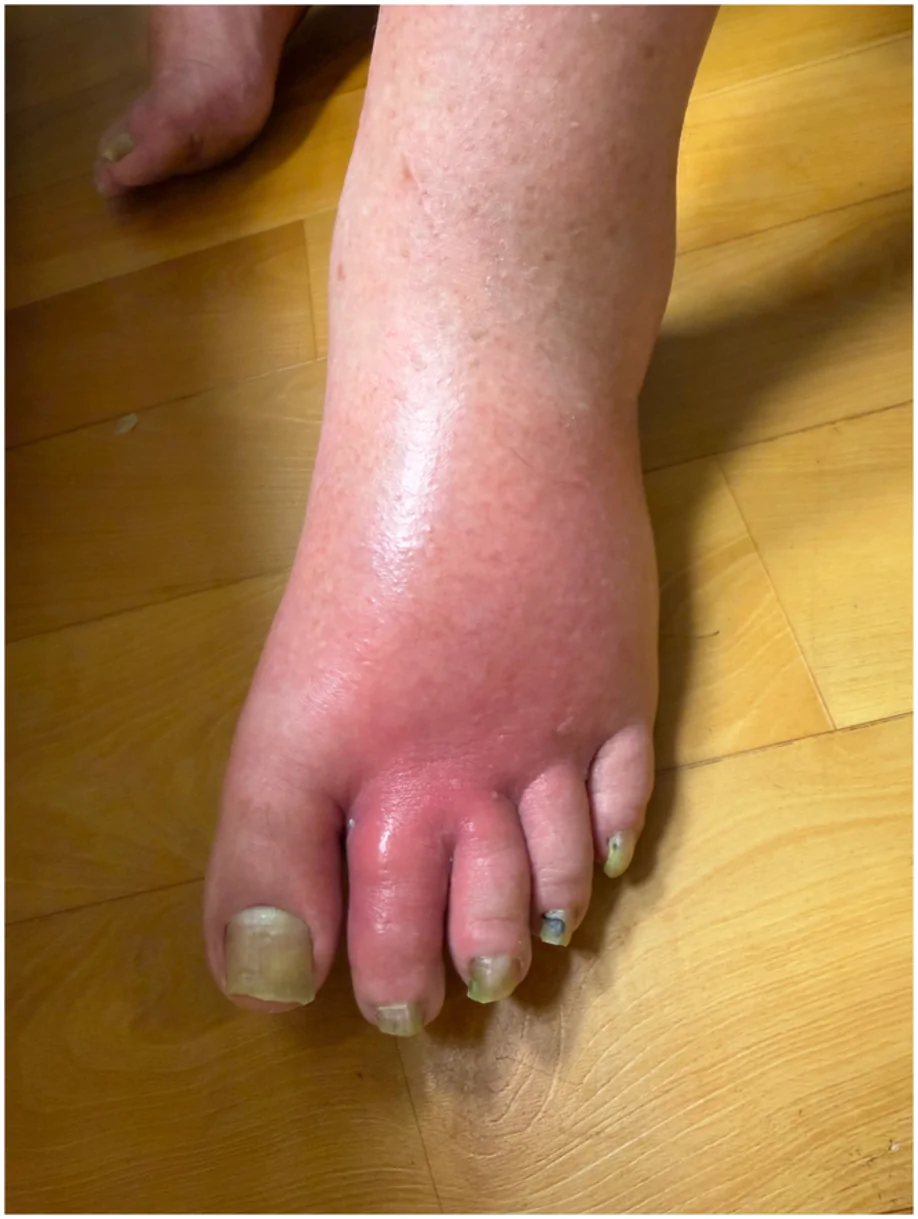
Left foot cellulitis with marked erythema and edema at the base of the second toe.

**Figure 2:**
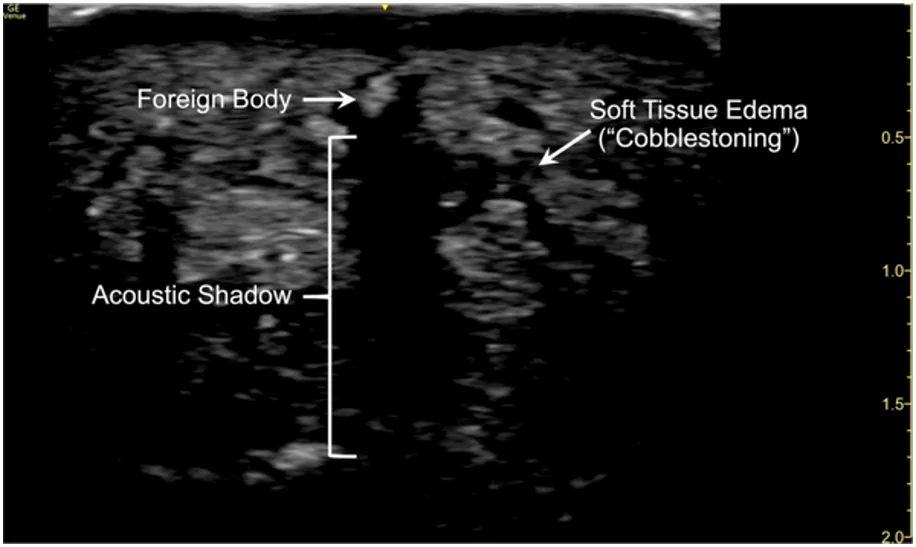
Ultrasound image from the plantar surface at the base of the second toe demonstrates a prominent vertical shadow from a retained foreign body at approximately 5 mm depth.

**Video 1:** Ultrasound image acquired with a high‐frequency, linear transducer reveals a prominent vertical shadow in the center of the image, which is cast by a foreign body in the superficial tissue at approximately 5 mm depth.

**Figure 3:**
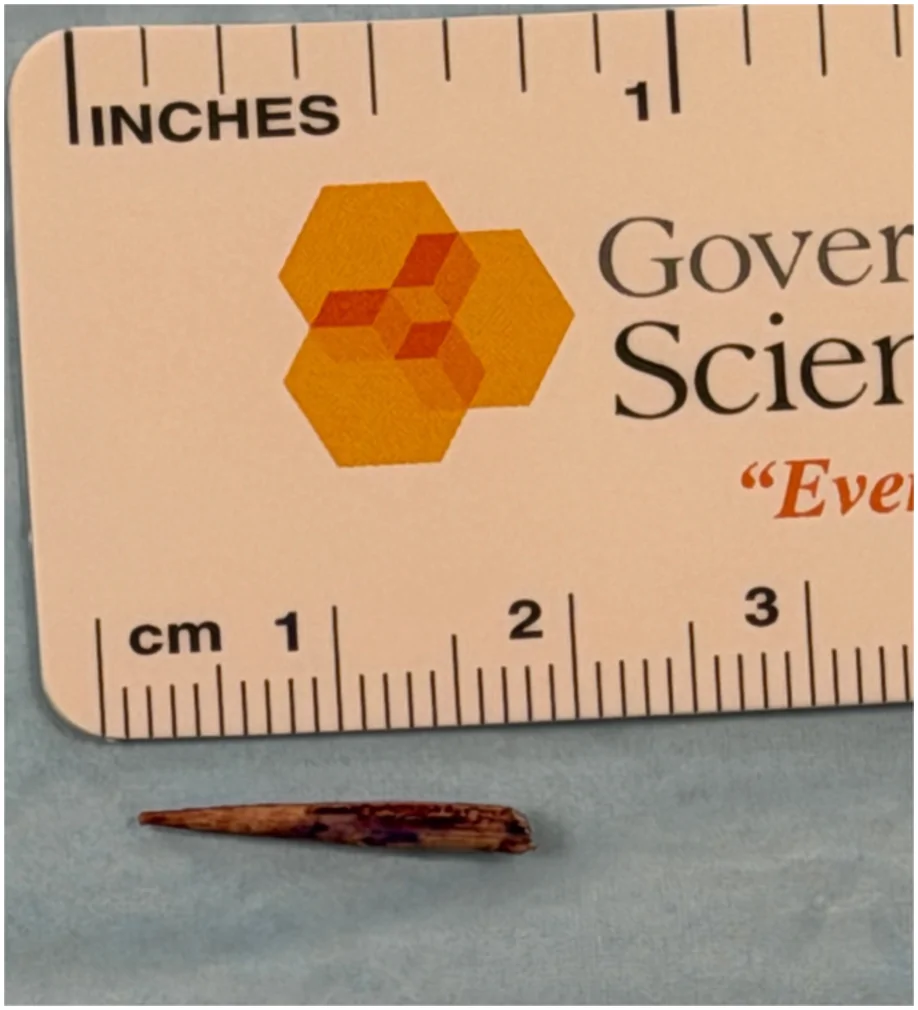
A 1.8 cm wooden toothpick was extracted by surgical exploration from the plantar surface of the left foot.

Approximately 80% of foreign bodies can be identified on plain radiographs, but wood is undetectable in 86% of patients.^1^ When considering additional imaging, CT imaging is not ideal, as wood can mimic the density of surrounding soft tissues. MRI also has low sensitivity for detecting retained wood, but it may be useful for chronic or deep‐seated fragments. In contrast, ultrasound can reliably detect wooden foreign bodies as small as 2.0–2.5 mm, most notably when retained in superficial soft tissues. Due to wood's echogenicity and distinctive shadowing, ultrasound is the preferred imaging modality.^2^

When suspecting a non‐metallic retained foreign body composed of wood, glass, or plastic, clinicians should consider using point‐of‐care ultrasound, as it has demonstrated high sensitivity as an imaging modality when compared to CT and MRI.

## CONFLICT OF INTEREST STATEMENT

The authors declare no conflicts of interest.

## DISCLAIMER

The contents of this publication do not represent the views of the US Department of Veterans Affairs or the US Government.

## Data Availability

The data that support the findings of this study are available on request from the corresponding author. The data are not publicly available due to privacy or ethical restrictions.
